# Squirmer hydrodynamics near a periodic surface topography

**DOI:** 10.3389/fcell.2023.1123446

**Published:** 2023-04-13

**Authors:** Kenta Ishimoto, Eamonn A. Gaffney, David J. Smith

**Affiliations:** ^1^ Research Institute for Mathematical Sciences, Kyoto University, Kyoto, Japan; ^2^ Wolfson Centre for Mathematical Biology, Mathematical Institute, University of Oxford, Oxford, United Kingdom; ^3^ School of Mathematics, University of Birmingham, Birmingham, United Kingdom

**Keywords:** microswimming, low Reynolds number flow, cell motility, confinement, surface topography

## Abstract

The behaviour of microscopic swimmers has previously been explored near large-scale confining geometries and in the presence of very small-scale surface roughness. Here, we consider an intermediate case of how a simple microswimmer, the tangential spherical squirmer, behaves adjacent to singly and doubly periodic sinusoidal surface topographies that spatially oscillate with an amplitude that is an order of magnitude less than the swimmer size and wavelengths that are also within an order of magnitude of this scale. The nearest neighbour regularised Stokeslet method is used for numerical explorations after validating its accuracy for a spherical tangential squirmer that swims stably near a flat surface. The same squirmer is then introduced to different surface topographies. The key governing factor in the resulting swimming behaviour is the size of the squirmer relative to the surface topography wavelength. For instance, directional guidance is not observed when the squirmer is much larger, or much smaller, than the surface topography wavelength. In contrast, once the squirmer size is on the scale of the topography wavelength, limited guidance is possible, often with local capture in the topography troughs. However, complex dynamics can also emerge, especially when the initial configuration is not close to alignment along topography troughs or above topography crests. In contrast to sensitivity in alignment and topography wavelength, reductions in the amplitude of the surface topography or variations in the shape of the periodic surface topography do not have extensive impacts on the squirmer behaviour. Our findings more generally highlight that the numerical framework provides an essential basis to elucidate how swimmers may be guided by surface topography.

## 1 Introduction

Ever since the studies of Robert Hooke and Antonie van Leeuwenhoek, it has been known that a drop of pond water contains countless microbes, many of which are motile, and indeed some can be lethal, such as *Naegleria fowleri*, the causative agent of amoebic meningitis. Even harmless motile microbes have attracted the attention of scientists for centuries, though more recently developments in nano- and micro-technology have also enabled fabrication of self-propelling artificial micro-robots and manipulation of their dynamics using microfluidic devices (Kherzi and Pumera, 2016). In laboratory experiments and observations, with both synthetic and biological swimmers, of the range of known control mechanisms, by far the most common is the influence of confining boundaries.

Nonetheless, the physical effects of walls on microswimmers can be subtle and have been extensively investigated theoretically, numerically, and experimentally in the past decade (Lauga and Powers, 2009; Elgeti et al., 2015). For instance, numerous biological microswimmers such as bacteria and sperm cells are known to accumulate near a flat wall boundary (Rothschild, 1963; Berke et al., 2008; Smith et al., 2009; Kantsler et al., 2013; Bianchi et al., 2017; Ohmura et al., 2018). Furthermore, motility near surfaces also has a functional role, for instance, biofilm formation and initial spread (Pratt and Kolter, 1998), as well as enhanced searching, which in turn is significant for fish egg fertilisation, where a sperm needs to enter the egg micropyle (Cosson et al., 2008). In addition, curved boundaries such as convex walls, corners, and obstacles are easily fabricated in microfluidic devices, which has motivated studies on the effects of such confinements both for biological microorganisms (Denissenko et al., 2012; Tung et al., 2014; Shum and Gaffney, 2015; Sipos et al., 2015; Ishimoto et al., 2016; Nosrati et al., 2016; Nishiguchi et al., 2018; Ostapenko et al., 2018; Rode et al., 2019; Yang et al., 2019) and artificial microswimmers (Takagi et al., 2014; Spagnolie et al., 2015; Liu et al., 2016; Yang et al., 2016; Wykes et al., 2017).

These curved boundaries and obstacles are typically larger than, or comparable to, the swimmer. If the structure on the wall boundary is smaller than the swimmer length scale, it may be considered a rough boundary instead of a completely flat surface. The impact of surface roughness has previously been considered *via* an asymptotically small amplitude of the surface topography in the presence of a spherical particle and a spherical microswimmer (Rad and Najafi, 2010; Assoudi et al., 2018), the so-called squirmer (Shaik and Ardekani, 2017).

The squirmer is a model microswimmer first proposed by Lighthill (1952) as a slightly deforming sphere and later corrected and used by Blake (1971) as a model of ciliate swimmers. This simple model is currently understood to provide qualitative predictions for a spherical biological microswimmer (Pedley, 2016; Pedley et al., 2016). In particular, a simplified version of the model, in which a rigid sphere can self-propel due to a given surface velocity slip profile, is known as the spherical tangential squirmer. This has been widely used as a simple mathematical model having a finite volume for studies on hydrodynamical aspects of microswimming such as nutrition uptake (Magar et al., 2003), cell–cell interactions (Ishikawa et al., 2006; Drescher et al., 2009), Janus particle motility (Spagnolie and Lauga, 2012; Ishimoto and Gaffney, 2013), collective dynamics (Evans et al., 2011; Zöttl and Stark, 2012; Delfau et al., 2016; Oyama et al., 2016), swimming in a non-Newtonian medium (Lauga, 2009; Zhu et al., 2012; Nganguia and Pak, 2018), and swimming near a wall (Llopis and Pagonabarraga, 2010; Spagnolie and Lauga, 2012; Ishimoto and Gaffney, 2013). The squirmer has also been studied in the context of confinement and obstacles such as the interior of a tube (Zhu et al., 2013), the presence of lattice-like multiple obstacles (Chamolly et al., 2017), or a curved and structured wall (Das and Cacciuto, 2019).

Investigations into the effects of small surface topography on microswimmers are, however, limited to the asymptotic analysis of rough surfaces or boundary features (Kurzthaler and Stone, 2021) such as curvatures with length scales that are much larger than those of the swimmer. The current study, therefore, aimed to bridge the gap between an asymptotically small amplitude of the surface roughness and large length scale curved boundaries. For periodic structures at this mesoscale, in particular, there is the prospect that the microswimmer may become oriented and guided by the surface, and we will numerically investigate the dynamics of a spherical tangential squirmer under these conditions. Such investigations are particularly motivated by recent studies of a colloidal microswimmer near a small surface topography (Simmchen et al., 2016), highlighting that a structured surface topography may be fabricated in a microfluidic device with the potential for utilisation in guiding microswimmers.

The very near-wall dynamics, at a separation of around 50 nm and less (Klein et al., 2003), typically depends on both hydrodynamic and steric interactions (Klein et al., 2003; Kantsler et al., 2013; Bianchi et al., 2017), and a short-range repulsive potential force is often utilised by modelling studies to ensure that simulated cells do not penetrate the walls (Spagnolie and Lauga, 2012). However, even a small difference in the repulsion function can alter swimmer behaviour (Lintuvuori et al., 2016; Ishimoto, 2017). Thus, in this initial study, we only focus on the hydrodynamic interactions and do not consider any additional steric interactions, contact mechanics, and charge effects.

This is motivated not only by the utility of the relative simplicity in this initial study but also for understanding the impact of hydrodynamic surface interactions where, despite these non-hydrodynamic forces, swimmer deposition is undesirable and thus of minimal interest, compared to topography guidance dynamics when deposition does not occur. It also entails that the results and conclusions of this study are not contingent on the details of contact mechanics and steric forces, which vary with surfaces and solutes (Klein et al., 2003). Thus, the numerical simulations are stopped just before the squirmer dynamics is influenced by the short-range dynamics on a very close surface approach. This short-range detail may be accommodated in later work together with many further refinements, such as incorporating more faithful representations of flagellated and ciliated swimmers (Ohmura et al., 2018; Ohmura et al., 2021).

The structure of this paper is as follows: Section 2 introduces the squirmer model and three different surface topographies. Section 3 discusses the numerical methods and their verifications. Section 4 presents the simulation results for the different surface topographies, followed by a discussion of the implications, in particular for microswimmer guidance *via* surface topography, in Section 5.

## 2 The squirmer

We consider the non-dimensional Stokes equations of the low Reynolds number flow, from which it follows for a velocity field ***u*** and a pressure field *p* that

∇p=Δuand∇⋅u=0. (1)

We impose the no-slip boundary condition on the swimmer and the wall, together with the force and torque balance equations for a swimmer with negligible inertia.

**FIGURE 1:**
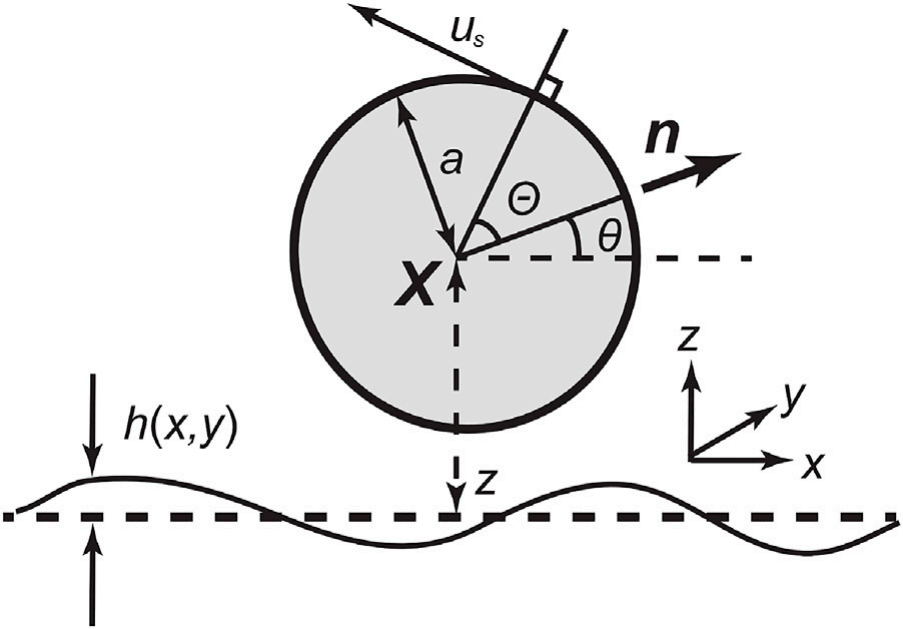
Schematic diagram of a spherical tangential squirmer, of radius *a* = 1, near a no-slip wall. Here, **X** denotes the centre of the spherical swimmer relative to a laboratory reference frame, with Cartesian coordinates (*x*, *y*, *z*) and *h*(*x*, *y*) is the height of the surface above its average midplane (dashed) at *z* = 0. Furthermore, *z* is overloaded and also represents the height of the swimmer centre above the midplane, with an analogous overloading of *x*, *y*. Whether *x*, *y*, *z* refer to coordinates or the overloaded definition ***X*** = (*x*, *y*, *z*) for the location of the squirmer centre will be clear from context. The unit vector ***n*** gives the orientation of the swimmer, which makes an angle *θ* relative to the mid-plane of the surface topography, and Θ is the local polar angle of a point on the swimmer’ surface relative to ***n***. The swimmer’s motility is driven by an axisymmetric tangential velocity of its surface, of size *u*_*s*_, and in the direction of increasing Θ, as detailed in the main text.

We first introduce the spherical tangential squirmer model. The no-slip boundary is imposed on a spherical swimmer of dimensionless radius *a* = 1, possessing an axisymmetric and tangential surface velocity (Ishikawa et al., 2006). The sphere centre is located at ***X*** = (*x*, *y*, *z*), and ***n*** denotes the unit vector of its orientation, as shown schematically in Figure 1, where coordinates, variables for the position of the squirmer centre, and the angles *θ*, Θ are defined using a diagram. Here, *θ* is the angle made by the swimming direction and the *xy* plane. In particular, Θ ∈ [0, *π*] denotes the polar angle relative to ***n***, and we impose an axisymmetric tangential velocity slip *u*_*s*_ on the squirmer, given by

$$u_{s}\left(\Theta \right)=\overset{\infty }{\underset{n=1}{\sum }}B_{n}V_{n}\left(\cos\Theta \right),$$ (2)

where *V*_*n*_ is a function derived from the Legendre polynomial *P*_*n*_(*x*) *via*

$$V_{n}\left(x\right)=\frac{2\sqrt{1-x^{2}}}{n\left(n+1\right)}\frac{dP_{n}\left(x\right)}{dx}.$$ (3)

The swimming velocity in free space is dictated by the first mode, with ***U*** = (2/3)*B*_1_ ***n*** (Lighthill, 1952; Blake, 1971). We fix *B*_1_ = 3/2 so that the squirmer swimming speed is set to be unity in free space, and we neglect the higher modes by setting *B*_*n*_ = 0 for *n* ≥ 3 so that the swimmer is subsequently fully characterised by the squirmer parameter *β* = *B*_2_/*B*_1_ (Ishikawa et al., 2006). In particular, and following convention, the swimmer is denoted as a pusher when *β* < 0, a puller when *β* > 0, and a neutral swimmer when *β* = 0 (Evans et al., 2011). The second mode, associated with the parameter *B*_2_, corresponds to the flow induced by the Stokes dipole. In particular, a cell with a trailing flagellum, such as an *E. coli* bacterium or a sperm cell, behaves as a pusher; cells with leading flagella, such as *Chlamydomonas* and *Leishmania* (Walker et al., 2019), are modelled as pullers; and cells possessing fore–aft symmetry, such as ciliates, behave as neutral swimmers (Evans et al., 2011).

**FIGURE 2:**
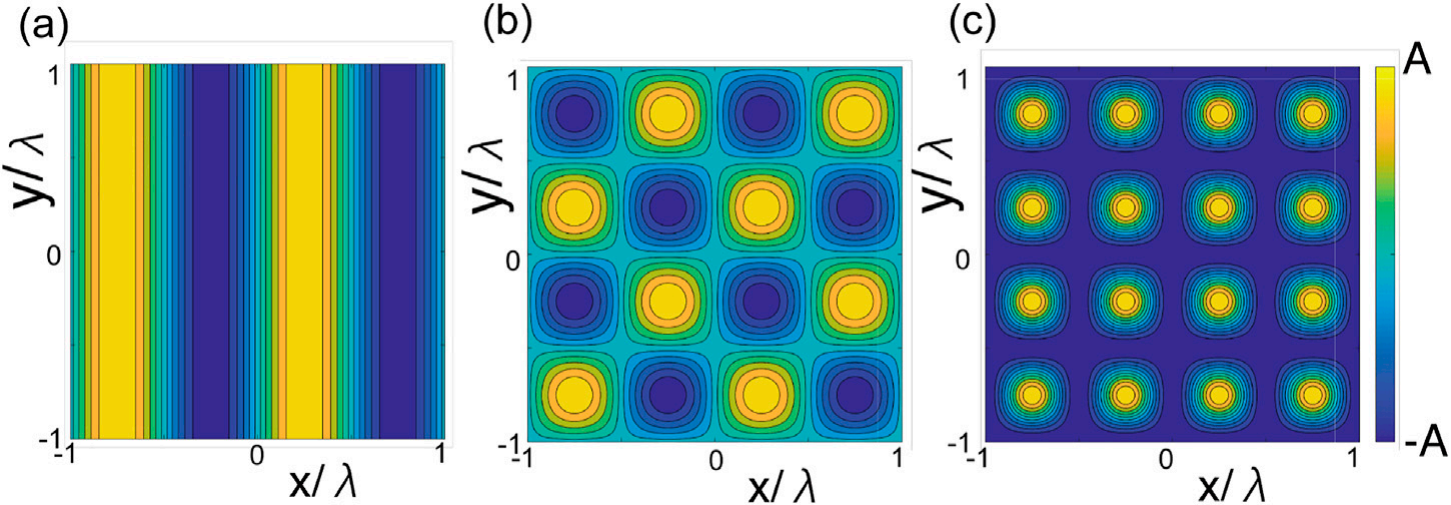
Illustrations of the surface topography functions *h*(*x*, *y*) considered in this study. **(A)** Singly periodic sinusoidal wave topography, **(B)** doubly–periodic sinusoidal wave, and **(C)** doubly–periodic peaks, respectively, associated with Eqs 4–6. The maximum and minimum heights, *h*(*x*, *y*), are +*A* and −*A* for all topographies.

Here, we focus on spherical tangential squirmers that swim stably near a flat surface. Thus, we consider puller squirmers with *β* ≳ 5, which are known to exhibit stable swimming near a flat surface (Ishimoto and Gaffney, 2013; Uspal et al., 2015). In particular, we examine their dynamics close to surfaces with structured topographies. The first of these topographies is a one-dimensional sinusoid defined by

$$h\left(x,y\right)=A\sin\left(kx\right),$$ (4)

where *A* is the amplitude and *k* = 2*π*/*λ* is the wavenumber, with *λ* denoting the wavelength (Figure 2A). The second topography is given by the doubly periodic sinusoid.

$$h\left(x,y\right)=A\sin\left(kx\right)\sin\left(ky\right),$$ (5)

as depicted in Figure 2B, and the third is given by

$$h\left(x,y\right)=A\left[2\sin^{2}\left(kx\right)\sin^{2}\left(ky\right)-1\right].$$ (6)

This final topography is shown in Figure 2C and presents doubly periodic peaks with highest and lowest heights of +*A* and −*A*, respectively, as in the previous two topographies. However, notably, the inter-peak wavelength is now halved, and the parameter *λ* = 2*π*/*k* no longer represents the wavelength since the sinusoidal functions are squared in Eq. 6. Throughout this study of the doubly periodic topographies, we focus on cases that are symmetrical in switching the *x* and *y*-directions.

We consider both the surface of the squirmer and the wall surface topography denoted by *S* and *W*, respectively, with the boundary conditions of the Stokes equations given by no-slip conditions on both boundaries. The surface velocity of the squirmer, denoted ***v*** (***x***), can be described by combining the squirmer linear velocity ***U*** and angular velocity **Ω**, together with its tangential surface velocity, ***u***_*s*_, of size given by Eq. 2 and in the axisymmetric tangential direction, as depicted in Figure 1. Hence, the no-slip condition entails the fluid velocity on the surface of the swimmer is given by

$$v\left(x\right)=U+\Omega \times \left(x-X\right)+u_{s}\left(x\right),\;\;\left(x\in \;S\right).$$ (7)

In contrast, the wall surface topography is assumed to be stationary, and we thus have

$$u\left(x\right)=0\;\;\left(x\in W\right).$$ (8)

## 3 Numerical methods

### 3.1 Nearest-neighbour regularised Stokeslet method

The dynamics of the squirmer has been computed using the nearest-neighbour regularised Stokeslet method (nnRSM) (Gallagher and Smith, 2018; Smith, 2018), and the numerical simulations have been performed based on the MATLAB code accompanied by Gallagher and Smith (2018), as we now summarise. The Stokes flow boundary integral equations for the single-layer formulation are given by (Pozrikidis, 1992)

$$u_{j}\left(x\right)=-\frac{1}{8\pi }\int_{S\cup W}S_{ij}\left(x,y\right)f_{i}\left(y\right)\;dS_{y}.$$ (9)

Here, *f*_*i*_(***y***) denotes the components of the surface traction and the integral kernel *S*_*ij*_ is the Stokeslet, which exhibits an integrable singularity as ***y*** → ***x***, with the surface integral well-defined. For numerical tractability, Cortez introduced a regularised Stokeslet (Cortez, 2001), which is the exact divergence-free solution to the Stokes flow equations with a spatially smoothed point force, and then approximated the boundary integral (Cortez et al., 2005) *via*

$$u_{j}\left(x\right)=-\frac{1}{8\pi }\int_{S\cup W}S_{ij}^{\epsilon }\left(x,y\right)f_{i}\left(y\right)\;dS_{y},$$ (10)

where $S_{ij}^{\epsilon }$ is the regularised Stokeslet and *ϵ* is the regularisation parameter. As the error in this approximation is *O*(*ϵ*), we recover the singular boundary integral once we take the limit of *ϵ* → 0. Following Cortez et al. (2005), we consider a regularised Stokeslet of the form,

$$S_{ij}^{\epsilon }\left(x,y\right)=\left\{\left(r^{2}+2\epsilon^{2}\right)\delta_{ij}+r_{i}r_{j}\right\}{\left(r^{2}+\epsilon^{2}\right)}^{-3/2},$$ (11)

where ***r*** = ***x*** − ***y***, *r* = | ***r*** |, and *δ*_*ij*_ represent Kronecker’s delta. The no-slip boundary conditions are simply given by enforcing ***u*** (***x***) = ***v*** (***x***) from Eqs. 7, 8 for boundary points in the integral Eq. 10. Since the squirmer is swimming freely, we also have the inertialess force and torque balance equations.

$$\int_{S}f\left(x\right)\;dS_{x}=0\;,\;\;\int_{S}\left(x-X\right)\times f\left(x\right)\;dS_{x}=0.$$ (12)

We then discretise the surface integrals (10), (12), by introducing the quadrature node positions ***x*** [*n*] and the associated weights *A*[*n*] for the discretised surface point *n* (*n* = 1, …, *N*), where *N* is the total number of surface points. The aforementioned surface integrals contain the product of ‘***f*** *dS*’ and we discretise the integral (Gallagher and Smith, 2018; Smith, 2018) *via*

$$\int •\;f_{j}\left(x\right)\;dS_{x}\approx \overset{N}{\underset{n=1}{\sum }}•\;g_{j}\left[n\right]A\left[n\right],$$ (13)

where the symbol, •, on the right-hand side, represents an arbitrary function of ***x***, evaluated at ***x*** [*n*], and *g*_*j*_[*n*] = *f*_*j*_(***x*** [*n*]).

**FIGURE 3:**
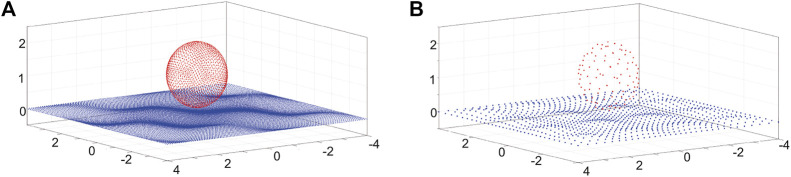
Illustration of the points representing the squirmer and surface topography, with the finer discretisation **(A)** used for the kernel and the coarser discretisation **(B)**, used for the surface traction. Discretisation points on the spherical squirmer surface are indicated by red dots. In contrast, blue discretisation points are shown for the doubly periodic surface topography of Figure 2B, with the size of the discretised surface given by *L* = 8 and the surface topography given by Eq. 5, as plotted in Figure 2B, with an amplitude of *A* = 0.1 and the wavelength given by *λ* = 2. Note that we here display the wall meshes after rescaling by the method described at the end of Section 3.

Continuing with the framework of Smith (2018), we introduce a second surface discretisation, ***x*** [*q*], (*q* = 1, …, *Q*) which corresponds to a more refined discretisation than used for the surface traction, with *N* ≪ *Q*, as illustrated in Figure 3. The regularisation error is *O*(*ϵ*), and this motivates taking relatively small values of *ϵ* in computations. The two discretisations enable an efficient numerical solution as the kernel, $S_{ij}^{\epsilon }$ , which can vary rapidly, and thus requires a finer discretisation, which would be inefficient if used for the surface traction, ***f***. In particular, the size of the dense linear system depends only on *N*, not *Q*. Thus, the cost of assembling the system is defined by *O*(*NQ*), whereas the cost of the direct solver is defined by *O*(*N* ^3^). Moreover, if the force and quadrature discretisations do not overlap, the quadrature error no longer diverges as *ϵ* → 0, and hence a less refined force discretisation in this framework is in general more accurate than if the two discretisations coincide (Gallagher et al., 2019).

The nearest-neighbour matrix, *ν*[*q*, *n*], is then defined separately for a swimmer and a wall as

$$\left\{\begin{array}{cc} 1\; & if\;n=\mathrm{arg min}|x\left[\hat{n}\right]-x\left[q\right]|\;\left(x\left[\hat{n}\right],x\left[q\right]\in S\right)\\ 1\; & if\;\;n=\mathrm{arg min}|x\left[\hat{n}\right]-x\left[q\right]|\;\left(x\left[\hat{n}\right],x\left[q\right]\in W\right)\\ 0\; & otherwise, \end{array}\right.$$ (14)

where arg min is the argument at which the minimum is attained over the set $\hat{n}\in \left\{1,\ldots ,N\right\}$ , and we use this matrix to interpolate the discretisation *via*

$$f_{j}\left(x\left[q\right]\right)dS\left(x\left[q\right]\right)\approx \overset{N}{\underset{n=1}{\sum }}\nu \left[q,n\right]g_{i}\left[n\right]A\left[n\right].$$ (15)

Combining Eqs 13, 15 and noting the total number of the points for the finer discretisation *Q*, we have

$$\int •\left(x\right)\;f_{j}\left(x\right)\;dS_{x}\approx \overset{N}{\underset{n=1}{\sum }}g_{j}\left[n\right]A\left[n\right]\overset{Q}{\underset{q=1}{\sum }}•\left(x\left[q\right]\right)\;\nu \left[q,n\right].$$ (16)

We use $•\left(x\right)=S_{ij}^{\epsilon }\left(x^{\prime },x\right)$ in Eq. 16 to discretise the boundary integral (10), and we use •(***x***) = 1 and •(***x***) = *ϵ*_*ijk*_(*x*_*k*_ − *X*_*k*_) in Eq. 13 for the force and torque balance relations (12), respectively.

In particular, to solve the squirmer trajectory, we first need to determine its velocity, ***U***, and angular velocity, **Ω**, which can then be integrated over time to determine the squirmer location and orientation. First, it should be noted that at a fixed point in time, the squirmer location and orientation are obtained from previous integration or from the initial conditions at the start of the simulation. Then, the discretisations of Eqs 10, 12, with ***u*** = ***v*** eliminated in terms of ***U***, **Ω,** and the known ***u***_*s*_ *via* Eqs. 2, 3, 7, 8, give 3*N* + 6 constraints for the 3*N* + 6 scalar unknowns associated with the unknown surface tractions at the *N* discretisation points and the unknowns ***U***, **Ω**. The resulting linear system is readily solved, provided that collocation points are unique, and we have a non-singular dense linear system that can be solved directly *via* standard methods.

As is the case for both singular and regularised versions of the boundary integral representation for flow around a constant volume body, the integral equation admits a gauge freedom ***f*** → ***f*** + *α* ***m***, where *α* is any constant and ***m*** is the surface normal pointing into the fluid (this can be observed by applying incompressibility and the divergence theorem to deduce that *∫*_*S*_ *S*_*ij*_ *m*_*i*_ *dS* = 0). In the absence of boundary conditions for traction, this freedom results in the pressure being determined only up to an additive constant in the exact problem. Discretisation results in an invertible matrix, and hence a unique (approximate) solution, because the discretised integral is no longer evaluated precisely to 0; moreover, the non-uniqueness of the continuum solution for the pressure is not dynamically important as it does not affect either the total force or moment on the swimmer.

### 3.2 Swimming in a free space

We first examine the numerical accuracy of the swimming velocity calculation for the squirmer in *free space*. The squirmer parameter is set to *β* = 0, and the exact swimming speed is | ***U*** | = 1, as detailed in the previous section. We have fixed the regularisation parameter *ϵ* = 0.001 and examined the impact of changing the discretisation refinement. In particular, with the total number of the points that form the squirmer surface given by $N=6n_{s}^{2}$ and $Q=6N_{s}^{2}$ for each discretisation (Gallagher and Smith, 2018; Smith, 2018), changes in both *n*_*s*_ and *N*_*s*_ have been examined. The results of Table 1 establish numerical parameters sufficient to obtain our desired relative error of around 1%. Finally, we also note that changes in *β* have not been observed to alter the swimming speed, as expected.

**TABLE 1:** Predictions for the free space swimmer speed. The exact speed is given by |*U*| = 1 and its numerical calculation is presented for refinements of both the discretisations used in the nearest–neighbour regularised Stokeslet method, where $N=6n_{s}^{2}$ and $Q=6N_{s}^{2}$ .

*ϵ*	*n*_*s*_	*N*_*s*_	| ***U*** |
0.001	4	10	1.0135
0.001	4	12	1.0017
0.001	4	14	1.0073
0.001	4	16	1.0153
0.001	4	18	1.0048
0.001	5	10	1.0291
0.001	5	12	1.0079
0.001	5	14	1.0098
0.001	5	16	1.0113

### 3.3 Swimming near a wall

Hereafter, we set the squirmer discretisation parameters to be (*n*_*s*_, *N*_*s*_) = (4, 18) and consider the squirmer near a no-slip wall. As previously studied by the boundary element method (Ishimoto and Gaffney, 2013), a strong puller tends to stably swim near a *flat* wall. We, therefore, choose *β* = 7 and set the initial location of the squirmer centre to be (0,0,1.15), with the initial orientation given by *θ* = −0.17*π*, which is effectively the initial angle of attack relative to the mid-plane of the surface topography, as can be seen from Figure 1.

**FIGURE 4:**
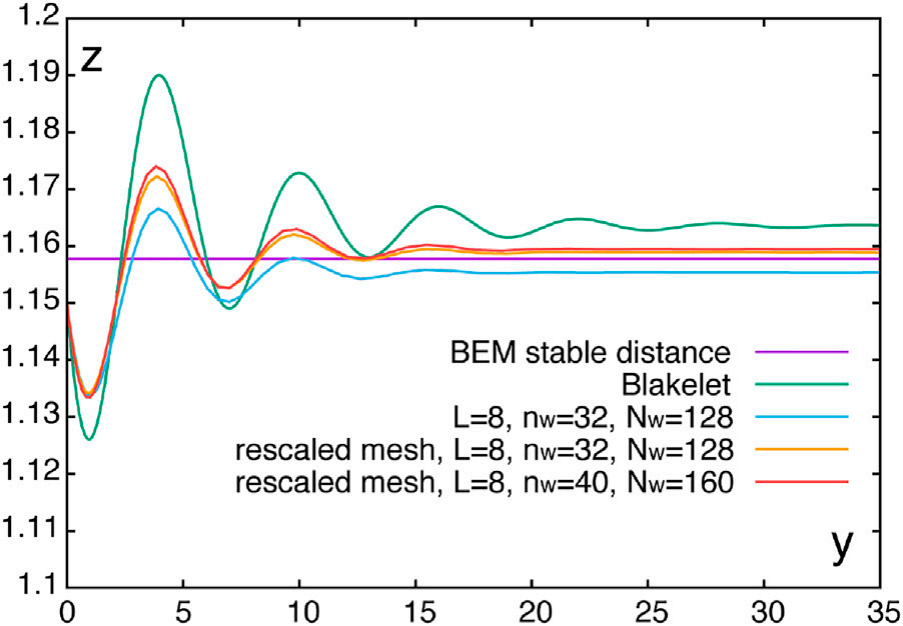
Swimming trajectories of the squirmer with a regularisation of *ϵ* = 0.001 and different discretisation parameters. Also plotted is the height of the stable fixed point obtained by the boundary element method using the singular Blakelet (Ishimoto and Gaffney, 2013), as labelled “BEM stable distance,” and a trajectory using a regularised Blakelet with the nearest-neighbour regularised Stokeslet method, as labelled by “Blakelet.”

We first use the regularised Blakelet (Ainley et al., 2008) in the nearest-neighbour regularised Stokeslet method and compare the simulation result with the stable distance obtained *via* the boundary element method using the singular Blakelet (Ishimoto and Gaffney, 2013), with the latter providing the stable separation distance z* ≈ 1.1578. As shown in Figure 4, these predictions of these two algorithms are in reasonable agreement.

We then consider a wall that is captured by an explicit discretisation of its surface rather than by use of the Blakelet, as implemented in the sperm simulation of Gallagher and Smith (2018). The wall is given by the *x*-*y* plane and represented by the square with a length of *L*, with its centre at the location of the projection of the squirmer centre, ***X***, onto the plane *z* = 0. Each side contains *n*_*w*_ and *N*_*w*_ points with equal separations for surface traction and kernel discretisations, respectively (Gallagher and Smith, 2018; Smith, 2018). Hence, the number of points on the surface *S* ∪ *W* are given by $N=6n_{s}^{2}+n_{w}^{2}$ and $Q=6N_{s}^{2}+N_{w}^{2}$ for the surface traction and kernel discretisations, respectively. An example of a swimming trajectory is plotted in Figure 4.

We then rescale the wall points to resolve the squirmer–boundary hydrodynamic interaction more efficiently by using the function

$$f:\left[-1/2,1/2\right]\to \left[-1/2,1/2\right],\;\;\;\;f\left(x\right)=\frac{1}{2}\tan\left(\frac{\pi x}{2}\right).$$ (17)

The equally discretised square of unit length

$$S=\left\{\left(x,y\right)\in \left[-1/2,1/2\right]\times \left[-1/2,1/2\right]\right\}$$ (18)

is mapped by this function, *via*

$$\left\{\left(f\left(x\right),f\left(y\right)\right);\left(x,y\right)\in S\right\},$$ (19)

and then dilated by the scale of *L*. The square obtained by this scheme more precisely represents the hydrodynamical interactions between the squirmer and the wall, as seen from the results labelled “rescaled” in Figure 4, which use this mapping. These trajectories in particular are sufficiently accurate for our purposes and very close to the prediction of the boundary element method (BEM) of the stable swimming height above the surface, which is exact to within discretisation error.

## 4 Results

**FIGURE 5:**
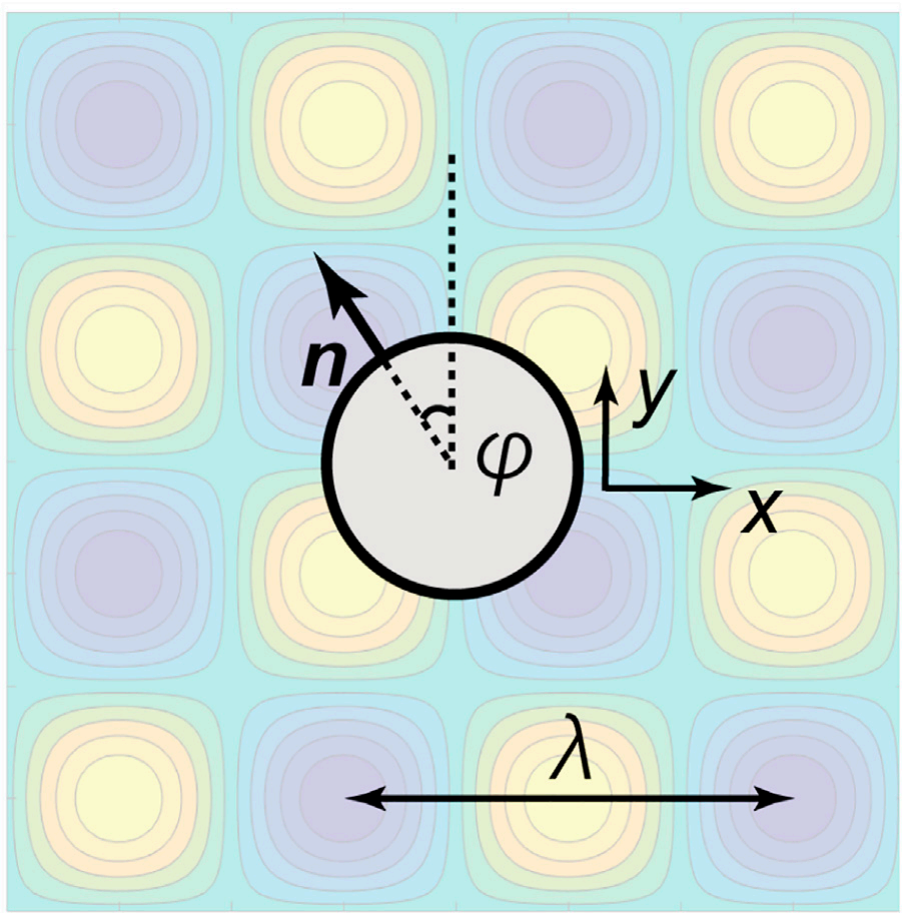
Bird’s eye view of the squirmer above the doubly periodic surface topography of Eq. 5, as depicted in Figure 2B, with *λ* = 4. The angle *φ* is defined to be the angle of the *x*–*y* projection of the squirmer orientation vector ***n*** from the *y*-axis, as illustrated, and thus gives the direction of the squirmer in the *x*-*y* plane.

In this section, we discuss the swimming trajectories of the squirmer near a surface with a structured periodic topography, as defined in Eqs. 4–6 and depicted in Figure 2. For all simulations presented, the initial height of the squirmer was fixed at *z* = 1.2, with the initial angle of attack given by *θ* = −0.17*π*. Furthermore, initial squirmer centre location coordinates of *x* = 0, *y* = 0, are set together with squirmer parameters of *B*_1_ = 3/2, *β* = 7, and a surface topography amplitude of *A* = 0.1, unless explicitly stated otherwise. Although the dynamics can change depending on the parameters and initial settings, we fix these variables to consider the stable behaviour and its modulation by surface topography, focussing on the impact of the initial orientation and topographic patterns. The surface topography wavelength and the initial orientation of the squirmer in the *x*–*y* plane, namely, *φ* in Figure 5, are thus varied extensively among the simulations and either stated or, in the case of *φ*, can otherwise be immediately inferred from the initial tangent angles of the trajectories in the presented plots.

### 4.1 Singly periodic sinusoidal topography

We start with the single-wave sinusoid topography given in Eq. 4 and Figure 2A. The initial location of the squirmer and the initial angle of attack are fixed at the simulation default initial values of ***X*** = (0, 0, 1.2) and *θ* = −0.17*π*, as stated previously, while the initial orientation of the squirmer in the *x*–*y* plane has been initially considered in detail with *φ* = 0.5*π* and then subsequently varied.

**FIGURE 6:**
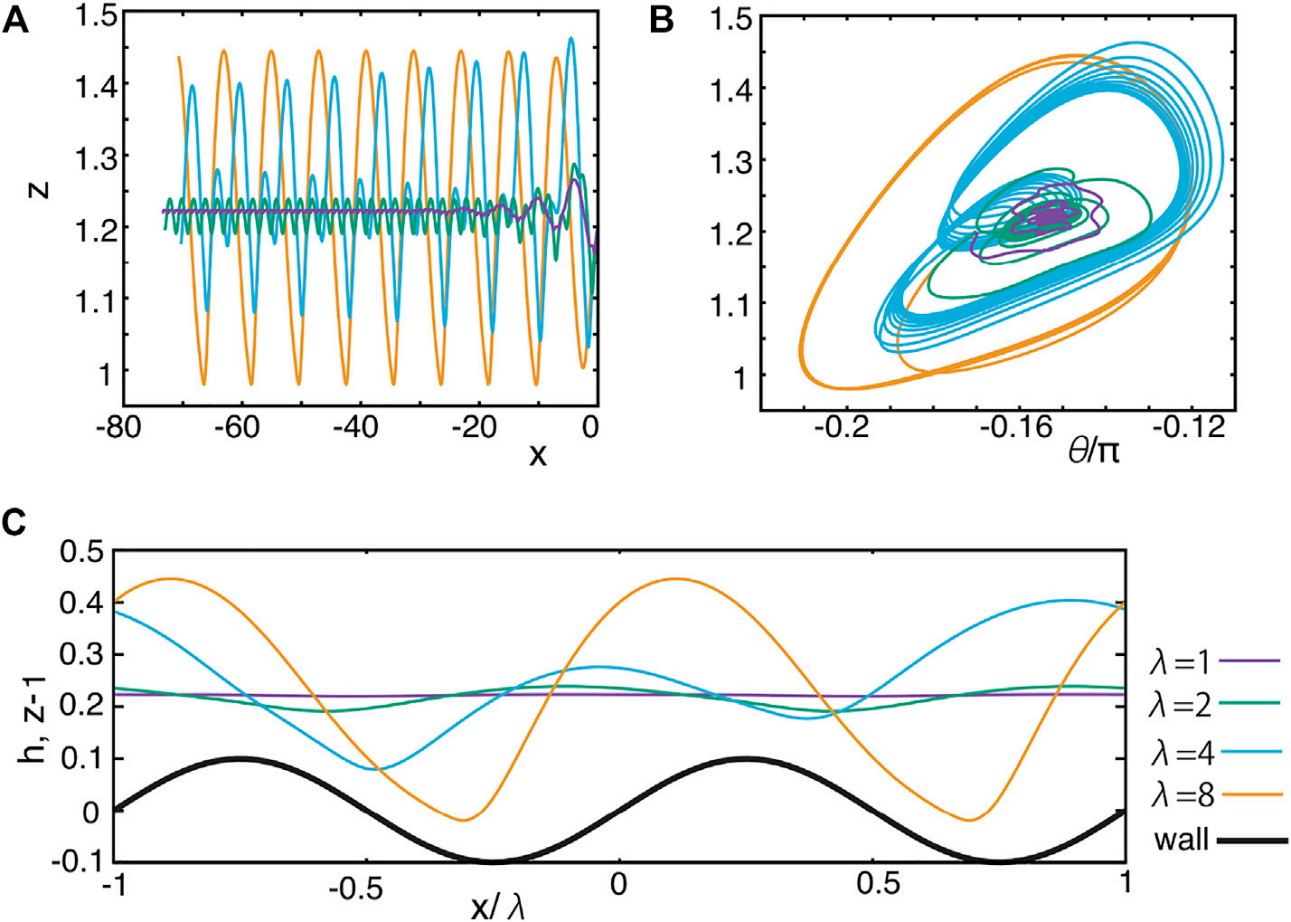
Dynamics of the squirmer swimming adjacent to the singly periodic sinusoidal topography of Eq. 4 and Figure 2A, with different wavelengths *λ* = 1, 2, 4, 8. The wave amplitude of the topography is fixed at *A* = 0.1. **(A)** Trajectories in the *x*–*z* plane. **(B)** Trajectories in the *θ*–*z* phase plane. **(C)** Horizontally rescaled and shifted *z* position for the last part of the simulation **(A)**, together with the topography function *h*.

Thus, we first consider a squirmer swimming parallel to the wave vector of the sinusoidal topography or equivalently along the *x*-axis. Fixing the initial orientation relative to the *x*–*y* plane *via* *φ* = 0.5*π*, swimming trajectories in the *x*–*z* plane are plotted in Figure 6A for different surface topography wavelengths *λ* = 1, 2, 4, 8. Corresponding trajectories in the *θ*-*z* phase plane are shown in Figure 6B. When the wavenumber *λ* is smaller (*λ* = 1, 2), the squirmer attains stable oscillatory swimming, but the oscillation in the *z*-axis is smaller than the surface topography amplitude, *A* = 0.1, highlighting that the topography only perturbs the stable position associated with swimming near a flat wall. This may be observed in Figure 6C, where the *z*-dynamics for the last part of the oscillating motion obtained in Figure 6A are shown relative to a horizontally rescaled and shifted axis *x*/*λ* together with the surface topography function *h*. However, as the wavelength is increased to *λ* = 4, *λ* = 8, the oscillatory motion then transitions to an amplitude that is larger than that of the surface topography, as can be seen in Figure 6C. In addition, one can observe that, with *λ* = 4, the wavelength of the *z*-component oscillations in the trajectory need not match that of the underlying surface topography, though in contrast, these two wavelengths do match for the trajectories with *λ* = 2 and *λ* = 8. Although the problem converges to the locally flat wall case in the large wavelength limit, the locally flat approximation does not hold for the range of *λ* we have examined, as may be inferred from the oscillatory motion in Figure 6C.

**FIGURE 7:**
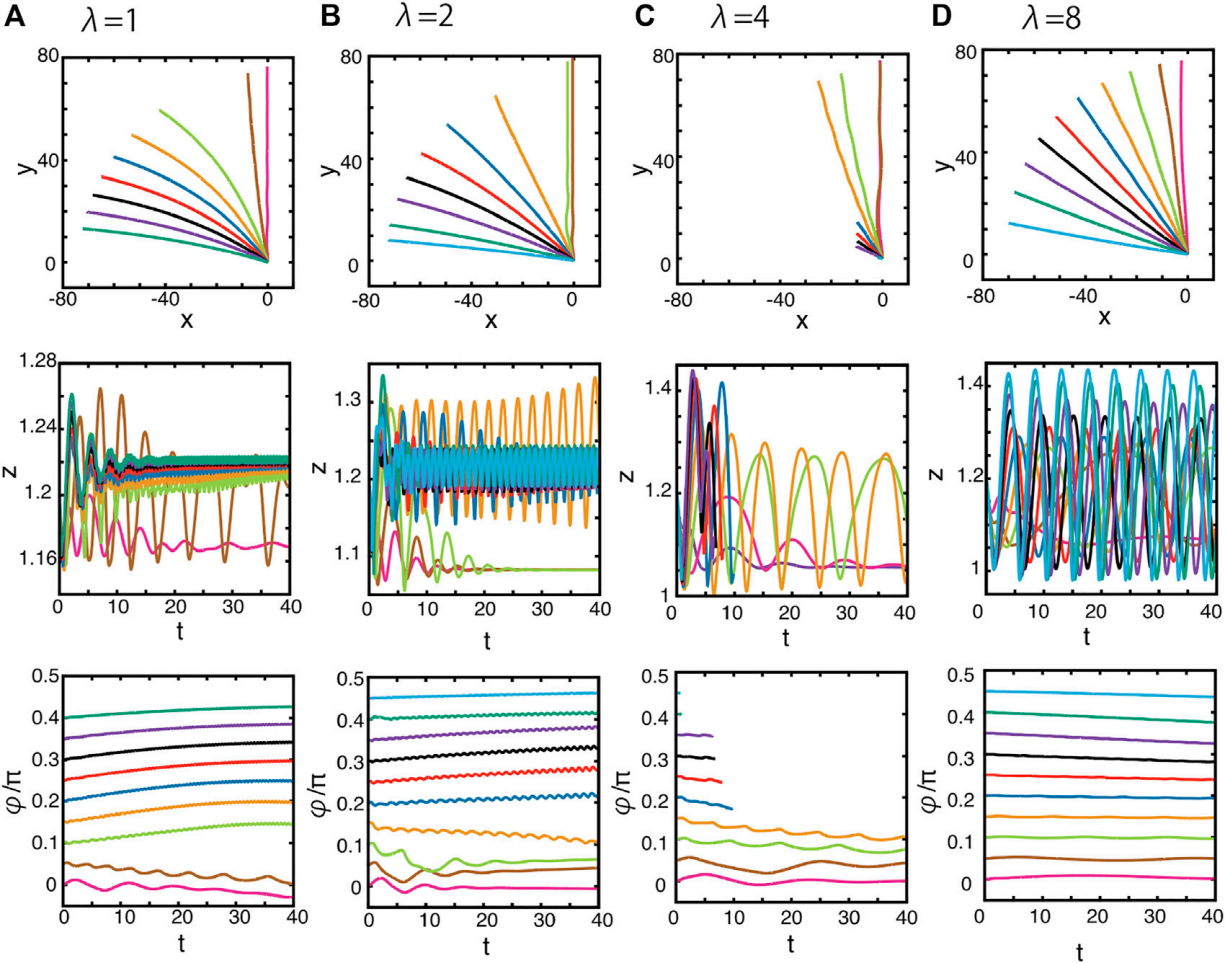
Dynamics of the squirmer near a surface with the singly periodic sinusoidal wave topography of Eq. 4 and Figure 2A. The surface topography amplitude is given by *A* = 0.1, and the wavelength is **(A)** *λ* = 1, **(B)** *λ* = 2, and **(C)** *λ* = 4, **(D)** *λ* = 8. (Top panels) the projections of the squirmer trajectories onto the *x*–*y* plane with different initial orientation angles, *φ*, as defined *via* Figure 5. These initial angles may be inferred from the initial tangents of the plotted projected trajectories. (Middle panels) the time evolution of the height of the centre of the squirmer, *z*. (Bottom panels) the time evolution of the orientation angle *φ*. Different colours index different initial values of the orientation angle, *φ*.

We then consider the squirmer dynamics with different initial values of *φ*, and thus different initial orientations relative to the *x*–*y* plane, while once again varying the wavelength of the singly periodic topography. Figure 7 shows the predicted trajectories and the orientations for the case with the surface topography amplitude *A* = 0.1 and wavelength *λ* = 1, 2, 4, 8, while considering various values of *φ*, from 0 to 0.5*π*. From the figure, one can observe that the squirmer tends to swim either parallel or perpendicular to the direction of the well, which is aligned along the *y*-axis, though the orientation angle *φ* need not always necessarily match the direction of motion. For instance, some trajectories in Figure 7 follow the topographical crest by swimming in the direction of the positive *y*-axis, with the orientation angle, *φ*, remaining very close to the initial value rather than aligning with the *y*-axis, even after a long time. Also, some further trajectories are attracted towards the negative *x*-axis, without the orientation angle *φ* evolving to reflect this change in swimming direction. Hence, overall drifting, that is, a misalignment between the swimming direction and swimmer orientation, can be induced by the squirmer–topography hydrodynamic interaction.

Notably, the swimming dynamics associated with an initial orientation angle of *φ* ≈ 0.05 with *λ* = 1, or *φ* ≈ 0.15 with *λ* = 2, is unstable, and the trajectories evolve towards the stable orientations of *φ* = 0, as seen in Figures 7A, B. Here, the stable swimming along the *y*-axis is accompanied by hydrodynamic capturing, with the squirmer moving along a trough of the surface topography. Furthermore, in both cases, the *z*-dynamics attains a stable oscillatory motion, as may be observed in Figures 6A, B. In both cases, the qualitative aspects of these features are unaltered when the amplitude *A* is decreased, though the timescale for the reorientation along one of the axes becomes longer.

Analogously, an increase in the topography wavelength to the intermediate value of *λ* = 4 entails that swimming oblique to the troughs and peaks of topography can be observed, as seen in Figure 7C. However, at this wavelength, the squirmer concomitantly undergoes extensive oscillations in the *z*-direction. Furthermore, the squirmer enters the near vicinity of the surface (Figure 7C) once it is no longer oriented approximately along the *y*-axis. This requires a consideration of surface mechanics to proceed. More precisely, in practical applications, surface mechanics would become relevant and would need to be added to the model. This is outside the detailed scope of the study and, hence, we stop the numerical simulation, thus also avoiding the numerically unreasonable spatial resolutions required for the associated fine-scale hydrodynamics.

We further increase the wavelength of the sinusoidal topography to *λ* = 8, with trajectories presented in Figure 7D, which on projection to the *x*–*y* plane, are essentially straight, regardless of the initial orientation angle *φ*. Hence, at larger wavelengths, the squirmer swims with a direction that is unaffected by the surface. Furthermore, the *z*-dynamics of the squirmer trajectory become more oscillatory as the topography wavelength increases, as observed previously in Figure 6, unless the squirmer is captured in the trough along the *y*-axis, in which case the *z*-dynamics converges to a stable position.

More generally, all of these observations highlight that even with a surface topography amplitude of *A* = 0.1, which barely visible, as highlighted by Figure 3, the squirmer’s behaviour is affected by the structured surface topography in a complex manner. In particular, the resulting trajectories are contingent on the details of the topography parameters and the squirmer orientation, especially once the topography wavelength is comparable to the squirmer size.

### 4.2 Doubly periodic sinusoidal topography

We now consider the squirmer dynamics near a surface with the doubly periodic wave topography, given by Eq. 5 and illustrated in Figure 2B. In the current setting, the topography breaks the translational symmetry in the *y*-direction, and hence the trajectories now also depend on the value of the initial *y* position of the squirmer centre. We first consider the squirmer starting with an orientation *φ* = 0.5*π* and an initial centre location coordinate of *y* = *λ*/4, with the other initial location coordinates and the initial angle of attack at the default values of *x* = 0, *z* = 1.2, and *θ* = −0.17*π*. Then, the squirmer moves along the −*x*-axis with similar dynamics in the *z*-direction to that displayed in Figure 6. In particular, the dynamics in the *z*-direction are only slightly perturbed when *λ* = 1, 2 but display larger oscillations when *λ* = 4, 8.

**FIGURE 8:**
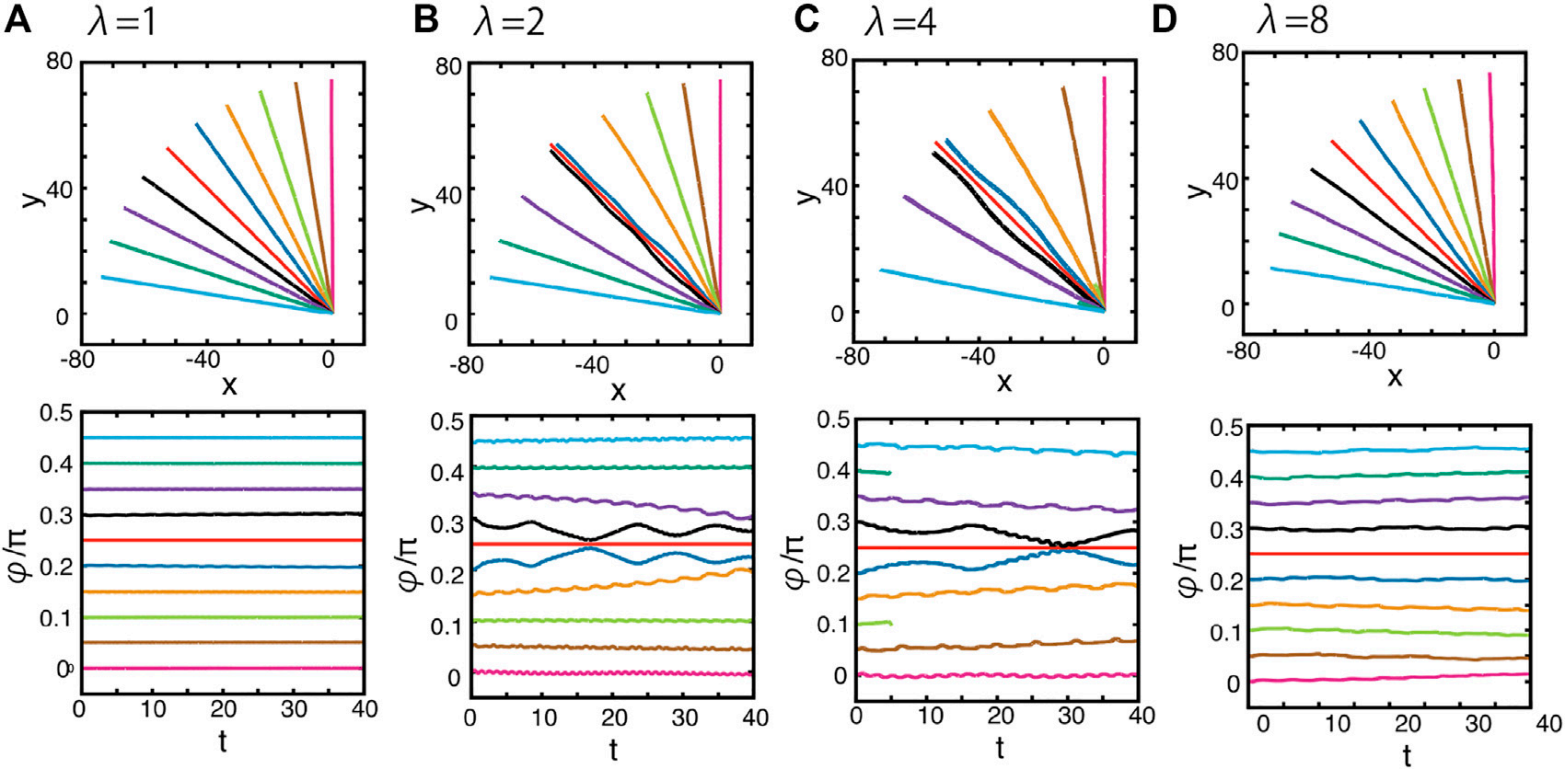
Dynamics of the squirmer near a surface with the doubly periodic sinusoidal wave topography of Eq. 5, as depicted in Figure 2B. The surface topography amplitude is given by *A* = 0.1, and the wavelength is **(A)** *λ* = 1, **(B)** *λ* = 2, **(C)** *λ* = 4, **(D)** *λ* = 8. (Top panels) the projections of the squirmer trajectories onto the *x*–*y* plane with different initial orientation angles, *φ*, as defined *via* Figure 5. These initial angles may be inferred from the initial tangents of the plotted projected trajectories. (Bottom panels) the time evolution of the orientation angle *φ*. Different colours index different initial conditions.

We then consider variations in the initial squirmer orientation angle *φ* that, as previously, entails the trajectories are no longer constrained to two spatial dimensions. The surface topography amplitude remains fixed at *A* = 0.1, and the initial height, location, and attack angle are at default values, while we consider variation in the orientation angle *φ* and the topography wavelength, which here is still given by *λ*. When *λ* = 1 (Figure 8A), the trajectories are not affected by the surface topography, as the trajectories are straight when projected on to the *x*–*y* plane, and the angle *φ* is constant in time. However, in the simulation with the wavelength *λ* = 2 (Figure 8B), some trajectories with *φ* ≈ *π*/4 are hydrodynamically captured near the bottom of the doubly periodic sinusoidal valley, whereas swimming outside of this region of initial orientation angles is not affected by the surface topography, as in the case of *λ* = 1.

These features can also be observed when we increase the wavelength to *λ* = 4 (Figure 8C). In contrast, for the larger wavelength of *λ* = 8, there is no evidence for an attracting trough of squirmer dynamics near the bottom of the topographic valley (Figure 8D). Together, these results highlight that the hydrodynamic attraction towards, and subsequently along, topographic valleys is not only limited but also only possible when the scale of the swimmer’s diameter is comparable with the length scale of the surface topography.

We then move to consider the final surface topography of doubly periodic peaks as introduced by Eq. 6 and displayed in Figure 2C. Trajectories with straight line projections onto the *x*–*y* plane can be observed when the squirmer’s initial location and orientation angle align along topography troughs or across topography crests. For example, given the default initial location of ***X*** = (0, 0, 1.2) and an initial orientation angle *φ* = 0, the squirmer swims with *φ* = 0 throughout time. In addition, these trajectories also exhibit nearly constant *z*-dynamics, though small *z*-oscillations are observed with amplitude ≲ 0.05 due to the topography, and this oscillation is further diminished as the topography wavelength increases. Furthermore, with the default initial location and an initial value of *φ* = 0.25*π*, or with initial values of ***X*** = (0, *λ*/4, 1.2) and *φ* = 0.5*π*, straight line *x*–*y* projected trajectories are also observed. Furthermore, in the *z*-direction, the squirmer behaviour changes from small oscillatory perturbations to larger amplitude *z*-oscillations as the wavelength increases, in direct analogy to the examples considered in detail with the previous topographies.

**FIGURE 9:**
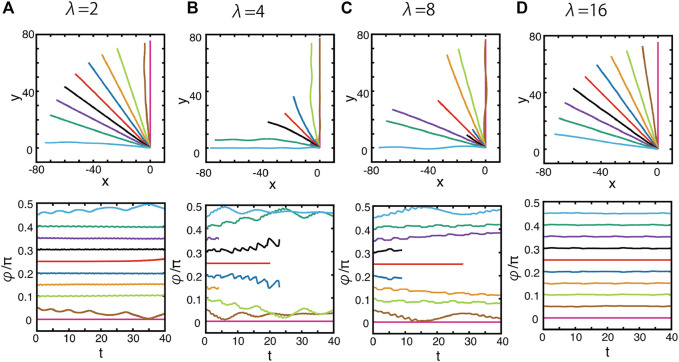
Dynamics of the squirmer near a surface with the doubly periodic sinusoidal wave topography of Eq. 6, as depicted in Figure 2C. The surface topography amplitude is given by *A* = 0.1, and the wavelength is unchanged from previous plots, but is no longer given by *λ* = 2*π*/*k* where *k* is the wavenumber of the sinusoidal functions in Eq. 6, since these functions are squared. Hence, to preserve wavelength at 1, 2, 4, 8 length units in the respective columns, we take **(A)** *λ* = 2, **(B)** *λ* = 4, **(C)** *λ* = 8, and **(D)** *λ* = 16 on moving from left to right across the figure. (Top panels) the projections of the squirmer trajectories onto the *x*–*y* plane with different initial orientation angles, *φ*, as defined *via* Figure 5. These initial angles may be inferred from the initial tangents of the plotted projected trajectories. (Bottom panels) the time evolution of the orientation angle *φ*. Different colours index different initial conditions.

For more general initial configurations of the squirmer, we have considered the three-dimensional behaviour of the resulting trajectories, with the simulation results plotted in Figure 9 for *λ* = 2, 4, 8, 16, observing the wavelength for this topography is *λ*/2, not *λ*. In the previous doubly periodic surface topography, swimming with the orientation angle *φ* = *π*/4 allowed the cell to move along a surface topography trough, noting that the prospect of drifting observed in the singly periodic topography of Figure 2A can be ruled out. In contrast, for the current case, an orientation of *φ* = *π*/4 moves across the surface topography peaks.

Again, the simulation results show that the squirmer is attracted by orientation angles corresponding to troughs in the surface topography, which here are *φ* = 0, *π*/2, for example. Furthermore, such topographic attraction is realised when the squirmer diameter is close to the characteristic length scale of the surface modulation, as seen in Figures 9A–C. However, again for the parameter regimes of Figures 9B, C, the swimmer can approach very close to the surface, and the trajectory simulations are halted as detailed surface dynamics are outside the scope of the study. Finally, we note that once the surface topography wavelength is increased further, with *λ* = 16 as presented in Figure 9D, all projected trajectories are straight lines, and the orientation angle *φ* is constant in time. Hence, at these larger wavelengths, the surface–swimmer interactions no longer influence the guidance of the squirmer.

## 5 Discussion

In this paper, we have numerically investigated the hydrodynamics of a puller spherical tangential squirmer near a surface with a singly or doubly periodic structured topography. In particular, the amplitude of the surface topography was an order of magnitude less than the squirmer size and a wavelength on the scale of the squirmer size. The simulated squirmer is known to be attracted to a stable separation from a flat wall, and a mesh-free regularised Stokeslet boundary element numerical scheme was demonstrated to accurately capture the dynamics induced by the subtle hydrodynamic interactions between a spherical tangential squirmer and a flat wall.

When the wavelength of the sinusoidal surface topography is smaller than the squirmer size, the perpendicular dynamics of the swimmer trajectory is a small amplitude oscillatory perturbation from the constant stable swimming height associated with a flat boundary. However, as the wavelength of the surface topography is increased, the squirmer acquires larger vertical, *z*-direction, oscillations with a wavelength that matches that of the topography at very large surface topography wavelengths, but not always at intermediate values (Figure 6C).

Furthermore, the squirmer movement in the horizontal, *x*–*y*, plane has been observed to be highly dependent on the detailed geometrical properties of the surface topography. We first considered a singly periodic sinusoidal surface topography. When the wavelength of the surface topography (Eq. 4) is not significantly larger than the squirmer diameter (*λ* < 4), the horizontal squirmer motion reorientates towards one of two stable directions, i.e., parallel and perpendicular to the wavevector of the surface topography, as seen in Figure 7. Furthermore, drifting can sometimes be observed, whereby the direction of motion differs slightly from the orientation angle, *φ*, as noted in Figure 7, for example.

At intermediate wavelengths (*λ* = 4) the squirmer can approach the surface. The detailed subsequent behaviour would be contingent on the near-surface physics, the detailed study of which is beyond the scope of this study. Once the surface topography wavelength is further increased, with *λ* = 8 sufficient so that the wavelength is four times that of the squirmer diameter, we observed that the horizontal motion is that of straight lines and surface induced guidance of the squirmer in the horizontal plane is lost.

For the doubly periodic surface topographies, the squirmer had the tendency to be locally guided to swim along surface topography troughs rather than over crests, though only when the squirmer diameter was comparable to the surface topography wavelength. However, such tendencies were weak and, at intermediate surface topography length scales, often accompanied by very close approaches to the surface where steric interactions would be important.

These qualitative features of the squirmer trajectories have also been observed when the squirmer parameter, *β* = *B*_2_/*B*_1,_ from Eq. 2, was varied within the range that induced stable swimming in the vicinity of a flat wall. In addition, changing the sinusoidal surface topography to a sigmoidal topography to represent fabricated surface wells in microfluidic devices did not induce a significant change in the qualitative features of the swimming trajectories. In turn, this evidences that squirmer swimming behaviours are influenced mainly by the length scale of the surface topography. In addition, we have also observed that the swimmer behaviour can be complex, especially when the swimmer is not aligned along the surface topography troughs or above the surface topography crests, though a limited local guidance to swim along the surface topography troughs has also been common.

There are considerable numbers of studies focussing on microswimmer dynamics near non-trivial geometrical structures such as curved obstacles (Nishiguchi et al., 2018; Das and Cacciuto, 2019), bumps (Simmchen et al., 2016; Yang et al., 2019), and maze-like micro-devices (Denissenko et al., 2012; Tung et al., 2014), but the length scale of the surface topography in the current study features much finer surface structures. We also note that the transitional vertical behaviours in the *z*-direction, from perturbations of the stable swimming height for a flat wall to topography-following motion at very large surface topography wavelength, necessitate a consideration of the finite-size amplitude of the surface topography. In particular, such behaviours highlight that the dynamics examined in this study requires larger-scale physics beyond the effective boundary conditions (Sarkar and Prosperetti, 1996; Kunert et al., 2010; Luchini, 2013) based on a very small amplitude surface roughness.

In the context of representing biological microswimmers such as spermatozoa and bacteria, which are pusher swimmers rather than pullers, hydrodynamic stable swimming occurs for prolate pusher tangential squirmers, but not spherical squirmers (Ishimoto and Gaffney, 2013). Moreover, the hydrodynamic interactions strongly depend on the swimmer morphology and beating pattern (Smith et al., 2009; Shum et al., 2010; Ishimoto and Gaffney, 2014; Walker et al., 2019). In turn, this highlights that detailed numerical studies are required to explore the surface dynamics for both prolate squirmers and more realistic microbiological swimmers near non-trivial surface topographies, including the prospect of a ciliated epithelium, modelled as a dynamic periodic boundary (Smith et al., 2008).

Furthermore, contact dynamics are also experimentally known to be significant for boundary accumulation behaviours of microswimmers (Kantsler et al., 2013; Bianchi et al., 2017) and to vary extensively with solutes and surfaces (Klein et al., 2003). However, the current study does not consider the detailed surface dynamics in the region very close to the boundary since its scope considers universal hydrodynamic interactions, rather than the boundary behaviours for a specific swimmer, solute, and surface. Even with a simple short-range repulsion, the details of the repulsive force can alter the swimmer dynamics (Lintuvuori et al., 2016; Ishimoto, 2017), while the contact mechanics reflect the specific biological and physical features of the system under investigation. A further generalisation to be considered in artificial colloidal microswimmers is the impact of sedimentation and gyrotaxis due to swimmer density heterogeneity and density offset from the fluid (Das et al., 2020), as well as the chemical and physical mechanisms that drive the colloidal particle (Uspal et al., 2015). Also, the swirling squirmer characterised by a rotlet–dipole singularity is known to exhibit circular motion near a boundary (Ishimoto and Gaffney, 2013), and the impact of the surface topography on such a swimmer warrants future investigations.

In summary, this investigation has used the nearest neighbour regularised boundary element method (Gallagher and Smith, 2018) to numerically explore the hydrodynamic interactions between a spherical tangential squirmer and a spatially oscillating surface topography with an amplitude that is an order of magnitude less than the squirmer size. In particular, a squirmer was investigated that swam with very simple dynamics close to a flat boundary, relaxing to a stable distance from the wall, and swimming in the direction of its orientation in the horizontal plane. However, even with small amplitude surface topographies, this squirmer’s dynamics has depended in a subtle and complex manner on the wavelength of the surface topography. We found that surface topographies could effect limited and local squirmer guidance towards topography troughs, in particular once the squirmer size is of the same order of magnitude as the surface topography wavelength. However, contact dynamics may also be induced at such wavelengths of the surface topography, especially if the initial squirmer orientation to the surface is not along topography crests or troughs. However, surface guided behaviours are robust to other aspects of the surface topography, such as reductions in the amplitude and changes in the shape of the surface topography waves. More generally, the framework enables these predictions to be made forming a basis for *in silico* experimentation of microorganisms and designing artificial micromachines.

## Data Availability

The raw data supporting the conclusions of this article will be made available by the authors without undue reservation. The data presented in this article is available from http://dx.doi.org/10.5287/ora-mzvppgob8.

## References

[B1] Ainley J. Durkin S. Embid R. Boindala P. Cortez R. (2008). The method of images for regularized Stokeslets. J. Comput. Phys. 4227, 4600–4616. 10.1016/j.jcp.2008.01.032

[B2] Assoudi R. Chaoui M. Feuillebois F. Allouche H. (2018). Motion of a spherical particle along a rough wall in a shear flow. Z. Angew. Math. Phys. 69, 112. 10.1007/s00033-018-1004-z

[B3] Berke A. P. Turner L. Berg H. C. Lauga E. (2008). Hydrodynamic attraction of swimming microorganisms by surfaces. Phys. Rev. Lett. 101, 038102. 10.1103/PhysRevLett.101.038102 18764299

[B4] Bianchi S. Saglimbeni F. Leonardo R. D. (2017). Holographic imaging reveals the mechanism of wall entrapment in swimming bacteria. Phys. Rev. X 7, 011010. 10.1103/physrevx.7.011010

[B5] Blake J. R. (1971). A spherical envelope approach to ciliary propulsion. J. Fluid Mech. 46, 199–208. 10.1017/s002211207100048x

[B6] Chamolly A. Ishikawa T. Lauga E. (2017). Active particles in periodic lattices. New J. Phys. 19, 115001. 10.1088/1367-2630/aa8d5e

[B7] Cortez R. Fauci L. Medovikov A. (2005). The method of regularized Stokeslets in three dimensions: Analysis, validation, and application to helical swimming. Phys. Fluids 17, 031504. 10.1063/1.1830486

[B8] Cortez R. (2001). The method of regularized Stokeslets. SIAM J. Sci. Comput. 23, 1204–1225. 10.1137/s106482750038146x

[B9] Cosson J. Groison A.-L. Suquet M. Fauvel C. Dreanno C. Billard R. (2008). Marine fish spermatozoa: Racing ephemeral swimmers. Reproduction 136, 277–294. 10.1530/REP-07-0522 18524881

[B10] Das S. Cacciuto A. (2019). Colloidal swimmers near curved and structured walls. Soft Matter 15, 8290–8301. 10.1039/c9sm01432b 31616894

[B11] Das S. Jalilvand Z. Popescu M. N. Uspal W. E. Dietrich S. Kretzschmar I. (2020). Floor- or ceiling-sliding for chemically active, gyrotactic, sedimenting janus particles. Langmuir 36, 7133–7147. 10.1021/acs.langmuir.9b03696 31986887 PMC7331144

[B12] Delfau J. B. Molina J. Sano M. (2016). Collective behavior of strongly confined suspensions of squirmers. EPL 114, 24001. 10.1209/0295-5075/114/24001

[B13] Denissenko P. Kantsler V. Smith D. J. Kirkman-Brown J. (2012). Human spermatozoa migration in microchannels reveals boundary-following navigation. Proc. Natl. Acad. Sci. U. S. A. 109, 8007–8010. 10.1073/pnas.1202934109 22566658 PMC3361448

[B14] Drescher K. Leptos K. C. Tucval I. Ishikawa T. Pedley T. J. Goldstein R. E. (2009). Dancing *volvox*: Hydrodynamic bound states of swimming algae. Phys. Rev. Lett. 102, 168101. 10.1103/PhysRevLett.102.168101 19518757 PMC4833199

[B15] Elgeti J. Winkler R. G. Gompper G. (2015). Physics of microswimmers—single particle motion and collective behavior: A review. Rep. Prog. Phys. 78, 056601. 10.1088/0034-4885/78/5/056601 25919479

[B16] Evans A. A. Ishikawa T. Yamaguchi T. Lauga E. (2011). Orientational order in concentrated suspensions of spherical microswimmers. Phys. Fluids 23, 111702. 10.1063/1.3660268

[B17] Gallagher M. T. Choudhuri D. Smith D. J. (2019). Sharp quadrature error bounds for the nearest-neighbor discretization of the regularized stokeslet boundary integral equation. SIAM J. Sci. Comput. 41, B139–B152. 10.1137/18m1191816

[B18] Gallagher M. T. Smith D. J. (2018). Meshfree and efficient modeling of swimming cells. Phys. Rev. Fluids 3, 053101. 10.1103/physrevfluids.3.053101

[B19] Ishikawa T. Simmonds M. P. Pedley T. J. (2006). Hydrodynamic interaction of two swimming model micro-organisms. J. Fluid Mech. 568, 119–160. 10.1017/s0022112006002631

[B20] Ishimoto K. Cosson J. Gaffney E. A. (2016). A simulation study of sperm motility hydrodynamics near fish eggs and spheres. J. Theor. Biol. 389, 187–197. 10.1016/j.jtbi.2015.10.013 26542943

[B21] Ishimoto K. Gaffney E. A. (2014). A study of spermatozoan swimming stability near a surface. J. Theor. Biol. 360, 187–199. 10.1016/j.jtbi.2014.06.034 25014474

[B22] Ishimoto K. Gaffney E. A. (2013). Squirmer dynamics near a boundary. Phys. Rev. E 88, 062702. 10.1103/PhysRevE.88.062702 24483481

[B23] Ishimoto K. (2017). Guidance of microswimmers by wall and flow: Thigmotaxis and rheotaxis of unsteady squirmers in two and three dimensions. Phys. Rev. E 96, 043103. 10.1103/physreve.96.043103 29347500

[B24] Kantsler V. Dunkel J. Polin M. Goldstein R. E. (2013). Ciliary contact interactions dominate surface scattering of swimming eukaryotes. Proc. Natl. Acad. Sci. U. S. A. 110, 1187–1192. 10.1073/pnas.1210548110 23297240 PMC3557090

[B25] Kherzi B. Pumera M. (2016). Self-propelled autonomous nanomotors meet microfluidics. Nanoscale 8, 17415–17421. 10.1039/c6nr06665h 27714185

[B26] Klein J. Clapp A. Dickinson R. B. (2003). Direct measurement of interaction forces between a single bacterium and a flat plate. J. Colloid Interface Sci. 261, 379–385. 10.1016/S0021-9797(03)00095-X 16256545

[B27] Kunert C. Harting J. Vinogradova O. I. (2010). Random-roughness hydrodynamic boundary conditions. Phys. Rev. Lett. 105, 016001. 10.1103/PhysRevLett.105.016001 20867466

[B28] Kurzthaler C. Stone H. A. (2021). Microswimmers near corrugated, periodic surfaces. Soft matter 17, 3322–3332. 10.1039/d0sm01782e 33630004

[B29] Lauga E. (2009). Life at high Deborah number. EPL 86, 64001. 10.1209/0295-5075/86/64001

[B30] Lauga E. Powers T. R. (2009). The hydrodynamics of swimming microorganisms. Rep. Prog. Phys. 72, 096601. 10.1088/0034-4885/72/9/096601

[B31] Lighthill M. J. (1952). On the squirming motion of nearly spherical deformable bodies through liquids at very small Reynolds numbers. Commun. Pure Appl. Math. 5, 109–118. 10.1002/cpa.3160050201

[B32] Lintuvuori J. S. Brown A. T. Stratford K. Marenduzzo D. (2016). Hydrodynamic oscillations and variable swimming speed in squirmers close to repulsive walls. Soft Matter 12, 7959–7968. 10.1039/c6sm01353h 27714374

[B33] Liu C. Zhou C. Wang W. Zhang H. P. (2016). Bimetallic microswimmers speed up in confining channels. Phys. Rev. Lett. 117, 198001. 10.1103/PhysRevLett.117.198001 27858454

[B34] Llopis I. Pagonabarraga I. (2010). Hydrodynamic interactions in squirmer motion: Swimming with a neighbour and close to a wall. J. Non-Newt. Fluid Mech. 165, 946–952. 10.1016/j.jnnfm.2010.01.023

[B35] Luchini P. (2013). Linearized no-slip boundary conditions at a rough surface. J. Fluid Mech. 737, 349–367. 10.1017/jfm.2013.574

[B36] Magar V. Goto T. Pedley T. J. (2003). Nutrient uptake by a self-propelled steady squirmer. Q. J. Mech. Appl. Math. 56, 65–91. 10.1093/qjmam/56.1.65

[B37] Nganguia H. Pak O. S. (2018). Squirming motion in a Brinkman medium. J. Fluid Mech. 855, 554–573. 10.1017/jfm.2018.685

[B38] Nishiguchi D. Aranson I. S. Snezhko A. Sokolov A. (2018). Engineering bacterial vortex lattice via direct laser lithography. Nat. Commun. 9, 4486. 10.1038/s41467-018-06842-6 30367049 PMC6203773

[B39] Nosrati R. Graham P. J. Liu Q. Sinton D. (2016). Predominance of sperm motion in corners. Sci. Rep. 6, 26669. 10.1038/srep26669 27211846 PMC4876399

[B40] Ohmura T. Nishigami Y. Taniguchi A. Nonaka S. Ishikawa T. Ichikawa M. (2021). Near-wall rheotaxis of the ciliate tetrahymena induced by the kinesthetic sensing of cilia. Sci. Adv. 7, eabi5878. 10.1126/sciadv.abi5878 34669467 PMC8528427

[B41] Ohmura T. Nishigami Y. Taniguchi A. Nonaka S. Manabe J. Ishikawa T. (2018). Simple mechanosense and response of cilia motion reveal the intrinsic habits of ciliates. Proc. Nat. Acad. Sci. U. S. A. 115, 3231–3236. 10.1073/pnas.1718294115 PMC5879680 29531024

[B42] Ostapenko T. Schwarzendahl F. J. Böddeker T. J. Kreis C. T. Cammann J. Mazza M. G. (2018). Curvature-guided motility of microalgae in geometric confinement. Phys. Rev. Lett. 120, 068002. 10.1103/PhysRevLett.120.068002 29481277

[B43] Oyama N. Molina J. J. Yamamoto R. (2016). Purely hydrodynamic origin for swarming of swimming particles. Phys. Rev. E 93, 043114. 10.1103/PhysRevE.93.043114 27176397

[B44] Pedley T. J. Brumley D. R. Goldstein R. E. (2016). Squirmers with swirl: A model for volvox swimming. J. Fluid Mech. 798, 165–186. 10.1017/jfm.2016.306 27795576 PMC5070036

[B45] Pedley T. J. (2016). Spherical squirmers: Models for swimming micro-organisms. IMA J. Appl. Math. 81, 488–521. 10.1093/imamat/hxw030

[B46] Pozrikidis C. (1992). Boundary integral and singularity methods for linearized viscous flow. Cambridge University Press.

[B47] Pratt L. Kolter R. (1998). Genetic analysis of *Escherichia coli* biofilm formation: Roles of flagella, motility, chemotaxis and type I pili. Mol. Microbiol. 30, 285–293. 10.1046/j.1365-2958.1998.01061.x 9791174

[B48] Rad S. H. Najafi A. (2010). Hydrodynamic interactions of spherical particles in a fluid confined by a rough no-slip wall. Phys. Rev. E 82, 036305. 10.1103/PhysRevE.82.036305 21230169

[B49] Rode S. Elgeti J. Gompper G. (2019). Sperm motility in modulated microchannels. New J. Phys. 21, 013016. 10.1088/1367-2630/aaf544

[B50] Rothschild L. (1963). Non-random distribution of bull spermatozoa in a drop of sperm suspension. Nature 198, 381. 10.1038/200381b0 14087906

[B51] Sarkar K. Prosperetti A. (1996). Effective boundary conditions for Stokes flow over a rough surface. J. Fluid Mech. 316, 223–240. 10.1017/s0022112096000511

[B52] Shaik V. A. Ardekani A. M. (2017). Motion of a model swimmer near a weakly deforming interface. J. Fluid Mech. 824, 42–73. 10.1017/jfm.2017.285

[B53] Shum H. Gaffney E. A. (2015). Hydrodynamic analysis of flagellated bacteria swimming in corners of rectangular channels. Phys. Rev. E 92, 063016. 10.1103/PhysRevE.92.063016 26764813

[B54] Shum H. Gaffney E. A. Smith D. J. (2010). Modelling bacterial behaviour close to a no-slip plane boundary: The influence of bacterial geometry. Proc. R. Soc. A Math. Phys. Eng. Sci. 466, 1725–1748. 10.1098/rspa.2009.0520

[B55] Simmchen J. Katuri J. Uspal W. E. Popescu M. N. Tasinkevych M. Sanchez S. (2016). Topographical pathways guide chemical microswimmers. Nat. Commun. 7, 10598. 10.1038/ncomms10598 26856370 PMC4748132

[B56] Sipos O. Nagy K. Leonardo R. D. Galajda P. (2015). Hydrodynamic trapping of swimming bacteria by convex walls. Phys. Rev. Lett. 114, 258104. 10.1103/PhysRevLett.114.258104 26197146

[B57] Smith D. Gaffney E. Blake J. Kirkman-Brown J. (2009). Human sperm accumulation near surfaces: A simulation study. J. Fluid Mech. 621, 289–320. 10.1017/s0022112008004953

[B58] Smith D. J. (2018). A nearest-neighbour discretisation of the regularized stokeslet boundary integral equation. J. Comput. Phys. 358, 88–102. 10.1016/j.jcp.2017.12.008

[B59] Smith D. J. Gaffney E. A. Blake J. R. (2008). Modelling mucociliary clearance. Respir. Physiol. Neurobiol. 163, 178–188. 10.1016/j.resp.2008.03.006 18439882

[B60] Spagnolie S. E. Lauga E. (2012). Hydrodynamics of self-propulsion near a boundary: Predictions and accuracy of far-field approximations. J. Fluid Mech. 700, 105–147. 10.1017/jfm.2012.101

[B61] Spagnolie S. E. Moreno-Flores G. R. Bartolo D. Lauga E. (2015). Geometric capture and escape of a microswimmer colliding with an obstacle. Soft Matter 11, 3396–3411. 10.1039/c4sm02785j 25800455

[B62] Takagi D. Palacci J. Braunschweig A. B. Shelley M. J. Zhang J. (2014). Hydrodynamic capture of microswimmers into sphere-bound orbits. Soft Matter 10, 1784–1789. 10.1039/c3sm52815d 24800268

[B63] Tung C.-K. Ardon F. Fiore A. G. Suarez S. S. Wu M. (2014). Cooperative roles of biological flow and surface topography in guiding sperm migration revealed by a microfluidic model. Lab. Chip 14, 1348–1356. 10.1039/c3lc51297e 24535032 PMC4497544

[B64] Uspal W. E. Popescu M. N. Dietrich S. Tasinkevych M. (2015). Rheotaxis of spherical active particles near a planar wall. Soft Matter 11, 6613–6632. 10.1039/c5sm01088h 26200672

[B65] Walker B. J. Wheeler R. J. Ishimoto K. Gaffney E. A. (2019). Boundary behaviours of l*eishmania mexicana*: A hydrodynamic simulation study. J. Theor. Biol. 462, 311–320. 10.1016/j.jtbi.2018.11.016 30465777 PMC6333917

[B66] Wykes M. S. D. Zhong X. Tong J. Adachi T. Liu Y. Ristroph L. (2017). Guiding microscale swimmers using teardrop-shaped posts. Soft Matter 13, 4681–4688. 10.1039/c7sm00203c 28466943

[B67] Yang F. Qian S. Zhao Y. Qiao R. (2016). Self-diffusiophoresis of janus catalytic micromotors in confined geometries. Langmuir 32, 5580–5592. 10.1021/acs.langmuir.6b01214 27186661

[B68] Yang J. Shimogonya Y. Ishikawa T. (2019). Bacterial detachment from a wall with a bump line. Phys. Rev. E 99, 023104. 10.1103/PhysRevE.99.023104 30934287

[B69] Zhu L. Lauga E. Brandt L. (2013). Low-Reynolds-number swimming in a capillary tube. J. Fluid Mech. 725, 285–311. 10.1017/jfm.2013.225

[B70] Zhu L. Lauga E. Brandt L. (2012). Self-propulsion in viscoelastic fluids: Pushers vs. pullers. Phys. Fluids 24, 051902. 10.1063/1.4718446

[B71] Zöttl A. Stark H. (2012). Hydrodynamics determines collective motion and phase behavior of active colloids in quasi-two-dimensional confinement. Phys. Rev. Lett. 108, 118101. 10.1103/PhysRevLett.112.118101 24702421

